# Enhancing kidney disease prediction with optimized forest and ECG signals data

**DOI:** 10.1016/j.heliyon.2024.e30792

**Published:** 2024-05-07

**Authors:** Muhammad Binsawad

**Affiliations:** Department of Information Systems, King Abdulaziz University, Jeddah, 21589, Saudi Arabia

**Keywords:** Electrocardiogram (ECG), Optimized forest, Chronic kidney disease (CKD), Feature extraction

## Abstract

To improve the early detection of Chronic Kidney Disease (CKD) utilizing electrocardiogram (ECG) data, this study explores the use of the Optimized Forest (Opt-Forest) model. Exploiting the possible relationship between kidney function and ECG data, we investigate Opt-Forest's performance in comparison to popular machine learning (ML) models. We evaluate Opt-Forest and find that it outperforms other options in CKD prediction based on many measures such as classification accuracy (CA), false positive rate (FPR), and true positive rate (TPR). In comparison to previous models, Opt-Forest has superior sensitivity and specificity, with a TPR of 0.787 and a low FPR of 0.174. With an accuracy of 78.68 %, a KS of 0.641, and a low RMSE of 0.174, Opt-Forest also demonstrates robustness in CKD prediction. This study demonstrates the potential of Opt-Forest to improve patient outcomes and medical diagnostics, as well as the usefulness of ECG data in enhancing early CKD diagnosis. Prospective research avenues to advance precision medicine in nephrology involve investigating deep learning methodologies and incorporating patient-specific data.

## 1 Introduction

Millions of individuals worldwide are afflicted by kidney disease, a common and sometimes fatal medical ailment that places a heavy strain on both patients and healthcare systems (Rady and Anwar, 2019). Effective treatment and management of kidney illnesses depend on timely and correct diagnosis (Alshebly, 2019) (McClellan, 2005). Utilizing electrocardiogram (ECG) data, which may provide important insights into a person's cardiovascular health, is a promising way to improve early identification and prediction of renal illnesses (Ghosh et al., 2020). ECG signals have complex patterns and information that go beyond cardiac health and are frequently used to identify heart-related illnesses. Given that kidney function is closely related to circulatory dynamics, the relationship between the cardiovascular and renal systems has long been understood. ECG signal changes may represent these underlying physiological changes, making them a potential source of prognostic data for renal disorders (Murat et al., 2020). In recent years, the area of medical diagnostics has seen a revolution thanks to machine learning (ML) models, which have proven to be extraordinarily effective at tasks requiring pattern recognition, classification, and prediction (Najafi Moghaddam Gilani et al., 2021). The investigation of various ML techniques to use ECG data for the prediction of renal illness has resulted from this (Aggarwal et al., 2022). Innovative models are still required, nevertheless, to fully use ECG information and provide improved forecast accuracy.

The problem at hand centers on the urgent need to improve the accuracy and dependability of kidney disease prediction. This disorder affects millions of people globally and places a significant cost on healthcare systems. The ability of ECG data to give insightful information about cardiovascular health still needs to be utilized, despite the need for early and accurate diagnosis for successful therapy and management. By adding the Optimized Forest (Opt-Forest) method, which was created especially for exploiting ECG data to predict renal illness, this research seeks to close this gap. The main issue to be solved is whether Opt-Forest can go past the constraints of conventional ML methods and considerably increase the extraction of pertinent data from ECG signals, hence improving the early detection and management of kidney disorders.

The significance of cardiovascular evaluation in kidney health is shown by the complex relationship between ECG signals and forecasts of renal illness. Modifications in the morphology of the ECG waveform, such as deviations in the ST segment, abnormalities in the T wave, and variations in the QRS complex properties, provide important information about the underlying renal failure. These anatomical alterations function as markers for cardiac strain, electrolyte abnormalities, and left ventricular hypertrophy, all of which are prevalent in patients with CKD. Furthermore, the therapeutic significance of ECG characteristics in predicting renal outcomes is further highlighted by the fact that ECG-derived indices such as heart rate variability (HRV) and QT interval prolongation offer prognostic information on autonomic dysfunction and arrhythmogenic risk. Predictive models that incorporate these ECG-based predictors improve diagnosis accuracy and allow for focused therapies and proactive risk stratification to reduce cardiovascular problems in patients with CKD. This improves overall outcomes and lowers morbidity and mortality.

The primary objective of this study is to assess the effectiveness of the Opt-Forest algorithm in comparison to other established machine learning techniques commonly used in medical diagnostics including Composite Hypercube on Iterated Random Projections (CHIRP), Decision Table-Naive Bayes (DTNB), Forest by Penalizing Attributes (FPA), Logistic Model Tree (LMT), Multilayer Perceptron (MLP), Naïve Bayes (NB), Support Vector Machine (SVM), and Random Forest (RF). When applied to ECG data for renal disease prediction, Opt-Forest, according to our hypothesis, would show improved predictive accuracy and resilience. The proposed model is compared (with the rest of the employed models based on diverse assessment measures including true positive rate TPR), false positive rate (FPR), recall, classification accuracy (CA), Receiver operating characteristic (ROC) area, Precision-Recall curve (PRC) area, Kappa Statistics (KS), and root mean squared error (RMSE). The major scientific contributions of this study are.

• Introducing the Opt-Forest method, which uses ECG data to identify CKD early.
• Acknowledgment of ECG data as a useful source for understanding renal health and its potential for enhancing CKD diagnosis.
• To find significant ECG features for CKD prediction, many feature selection algorithms are being investigated (e.g., Harmony Search, Best First Search, and Particle Swarm Optimization (PSO)).
• Review of the entire performance, proving the advantage of Opt-Forest in terms of precision, and error rate.
• The importance of early CKD identification in the clinical setting and its effects on patients with renal and cardiovascular problems are emphasized.
• Contribution to the improvement of patient outcomes through early CKD treatment and the advancement of medical diagnostics.

This work presents the Opt-Forest method, a novel approach that uses ECG data to predict CKD. By using cutting-edge feature selection techniques like Harmony Search and Particle Swarm Optimization, it pioneers the use of ECG data for CKD diagnosis. It highlights early CKD diagnosis by comparison analysis with well-established machine learning algorithms, offering promising advances in medical diagnostics and better patient outcomes.

The rest of this paper is organized as follows: The literature reviewed in CKD and ECG signal analysis is summarized in Section 2. The Opt-Forest algorithm and research design are described in Sections 3, 4 respectively. Section 5 includes the experimental findings and a comparison of Opt-Forest with other used models. Section 6 outlines the paper's conclusions.

## 2 Literature study

To give patients more informed and specialized treatment, modern healthcare systems are improved with advanced components like ML, data mining, and artificial intelligent (AI). Using ML approaches, a team of internists and nephrologists developed a realistic strategy for the Kidney Disease Outcomes Quality Initiative (KDOQI) to help primary care physicians diagnose and manage patients with chronic kidney disease. Chronic kidney disease is defined as having a glomerular filtration rate (GFR) of 60 ml/min/1.73 m^2^ and/or indications of renal impairment for at least three months (Gudeti et al., 2020). Urinary albumin-creatinine ratio (UACR) and estimated glomerular filtration rate (eGFR) based on blood creatinine concentration are the two tests for chronic kidney disease that are most often employed in clinical settings. The estimated glomerular filtration rate and albuminuria should be assessed in people with diabetes and/or hypertension, but not in the general public (Battineni et al., 2020).

Healthcare entails identifying, avoiding, and treating medical problems while pursuing affordable yet efficient fixes. A key component of doing this is machine learning (ML). Clinical and claims data are examples of data types used in healthcare. These data categories are the main emphasis of this article's analysis of 11 chronic illnesses, including renal disease, osteoporosis, and arthritis. By comparing several diagnostic methods for each disorder according to their clinical relevance, ML techniques are used to investigate the relationships between these illnesses and the tests employed to diagnose them (Sawhney et al., 2023). Nusinovici et al. (Nusinovici et al., 2020) employed fundamental clinical indicators to evaluate the efficacy of machine learning (ML) algorithms in predicting the risk of cardiovascular diseases (CVDs), chronic kidney disease (CKD), diabetes (DM), and hypertension (HTN). It examined five ML models (neural network, SVM, RF, gradient boosting machine, and k-nearest neighbor) with Logistic Regression (LR) using data from 6762 Asian individuals. For CKD and DM prediction, logistic regression worked best, whereas neural networks and support vector machines performed best for CVD and HTN.

Revathy et al. (Revathy et al., 2019) address the critical challenge of predicting CKD. To analyze and forecast CKD based on the data at hand, they use a variety of ML models. The paper gives a brief review of several ML techniques, but its main emphasis is on performance assessment and comparative analysis. Based on criteria including accuracy, sensitivity, specificity, and area under the receiver operating characteristic curve (AUC-ROC), the study's findings include the development of the most efficient ML model for CKD prediction. The authors emphasize the potential for improving healthcare outcomes via the use of predictive models as they draw to a close by underlining the relevance of ML in early CKD prediction. Author in [1], employed ML approaches are investigated for the early identification of chronic kidney disease (CKD). NBTree, J48, SVM, LR, MLP, NB, and CHIRP were seven ML techniques examined to categorize CKD patients from a dataset. With a remarkable accuracy of 99.75 %, CHIRP beat other approaches in the study, demonstrating its efficacy in the early identification of CKD. Elhoseny et al. (Elhoseny et al., 2019) used density-based feature selection (DFS) and ant colony-based optimization (D-ACO) to create an intelligent healthcare system for CKD. The D-ACO classification method is then implemented after the system first uses DFS to remove duplicate features. On a benchmark CKD dataset, the D-ACO algorithm's performance was assessed. The outcomes showed that, even with a smaller dataset, the algorithm significantly improved classification accuracy when compared to previous approaches.

Early diagnosis of CKD may allow the patient to take the required medical actions to perhaps slow down or even reverse the course of the condition. Physionet Database (www.physionet.org) the publicly available databases PTB (for renal patients) and Fantasia (for healthy persons) provided the digitalized ECG data for this model, and afterward, additional data from the same web source was utilized to test the framework. The testing strategy yielded good findings since the algorithm could tell who had a renal illness and who did not because it could differentiate between the two groups of people. We discovered a degree of precision of 97.6 % in our research, which was the highest employing both parameters QT and RR interval. This precision is more than the accuracy discovered when using only one of the features (Singh and Krishnan, 2023), (Rahman, 2019). According to the research of E. P. B. Mulia et al. renal illness may be identified by looking at the patient's ECG data using predictive ML classification models. Recent research and studies have shown that individuals with renal disorders start to have cardiac problems, also known as Cordial Renal Syndrome (CRS), which, in the latter stages of their condition, can cause a sudden cardiac arrest (Mulia et al., 2021). Patients who have cardiovascular problems can use this model to determine whether or not their kidneys have been affected because chronic kidney disease and cardiovascular disorders are linked (Kusuma et al., 2020).

Evaluation of overall related work is presented in Table 1.

**Table 1:** Evaluation of the related work.

Study/Approach	Pros	Cons
Gudeti et al., 2020	Provides a realistic strategy for diagnosing and managing CKD.	Focuses primarily on diagnosis and management strategies rather than predictive modeling.
Sawhney et al., 2023	Utilizes ML techniques to investigate relationships between diagnostic methods and chronic illnesses.	Does not specifically focus on CKD prediction or utilize ECG data.
Nusinovici et al., 2020	Evaluates efficacy of ML algorithms in predicting cardiovascular diseases (CVDs), CKD, diabetes, and HTN.	Lack of focus solely on CKD prediction using ECG data; may not account for specific nuances of CKD prediction.
Revathy et al., 2019	Addresses the challenge of predicting CKD and compares various ML models.	May not explore feature selection techniques to optimize model performance; lacks focus on ECG data utilization.
[1]	Investigates ML approaches for early identification of CKD using various techniques.	Focuses on early identification rather than precise prediction; may not utilize feature selection or optimization methods.
Elhoseny et al., 2019	Utilizes density-based feature selection and ant colony-based optimization for CKD prediction.	May not specifically address ECG data utilization or provide comparative analysis with other feature selection techniques.
Singh and Krishnan, 2023; Rahman, 2019	Propose predictive ML classification models for identifying renal illness using ECG data.	Lack comprehensive evaluation of model performance or comparison with other approaches; may not address specific challenges in CKD prediction.
Mulia et al., 2021	Highlights cardiac problems in renal disorder patients and proposes ML models for detection.	Focuses on detection rather than precise prediction; may not provide detailed methodology or comparison with existing predictive models for CKD.

Compared to current techniques, the Optimized-Forest algorithm provides several benefits. To improve the prediction of CKD using ECG signal data, a suggested model known as the Optimized-Forest method combines sophisticated feature selection and model optimization approaches. By utilizing the CfsSubsetEval attribute evaluator in conjunction with Harmony Search, Best First Search, and Particle PSO techniques, the algorithm determines which features from the ECG dataset are most informative, hence minimizing redundancy and guaranteeing predicting accuracy. By adding a random element and prioritizing high-quality trees, the Optimized-Forest repeatedly improves decision tree subforests and maximizes ensemble accuracy. A wide range of evaluation measures, including as TPR, FPR, recall, ROC area, PRC area, KS, and RMSE, are used to thoroughly examine the model's performance and provide a detailed assessment of prediction accuracy and robustness. In order to demonstrate the Optimized-Forest's superiority and effectiveness in CKD prediction using ECG data, it is also evaluated against traditional machine learning models that are often employed in the field.

## 3 Optimized-Forest

Implementation of the "Optimized-Forest" algorithm, which was first released in [2]. This algorithm creates a decision forest and then uses a Genetic Algorithm (GA) to choose the best subforest inside it. Infusing high-quality trees as the beginning population for GA is the major contribution of the suggested forest pruning (i.e., subforest selection) approach. In our case, GA is a class of computational framework inspired by evolution [3]. John H. Holland developed GAs, which imitate natural evolution in computers [4]. Using chromosomes, GAs encode possible solutions. Starting with a population of chromosomes at random, the process involves genetic operations like crossover and mutation to produce a new population. The best-performing chromosomes are kept for future reproduction after periodic reviews which are produced by the GA. This work adds a fresh contribution to early population selection to the GA structure. The detailed steps are presented in algorithm 1.

**Table:** 

Algorithm 1
**Input:** - Population size: |P| = 20 -Number of iterations: J -Number of trees in a forest: T = 100 -Stratum sizes: |S1|, |S2|, |S3| -Probability of selecting an individual chromosome in roulette wheel selection: p(Crr) -Number of trees to select for an individual chromosome: M **Initialization:** Create an empty current population Pcurr Create an empty temporary population PTempCurr Create an empty modified population PMod Initialize CrS FBest as the best chromosome found so far **For j = 1 to J (Iterations):** **Step 1:** Initial Population Selection Initialize PCurr with 20 chromosomes as follows: −10 chromosomes prioritize good-quality trees (odd chromosomes) −10 chromosomes accumulate trees randomly (even chromosomes) **For odd chromosomes:** -Perform stratiﬁed sampling with three strata: S1, S2, S3 -Randomly select M from S1, S2, and S3 using Disproportionate Stratiﬁed Sampling (DSS) **For even chromosomes:** -Randomly select M trees from the forest **End Step** **Step 2:** Crossover and Mutation Select chromosome pairs for crossover using roulette wheel selection and apply 1-point crossover. Perform 1-bit flipping mutation on offspring chromosomes. **End Step** **Step 3:** Elitist Operation Duplicate chromosomes to PTempCurr and store the best chromosome as CrCurrBest. Apply crossover to create PMod. Compare CrModBest with CrS FBest, update if better. Replace the worst chromosome in PMod with CrCurrBest if it's better. Update CrCurrBest from PMod. **End Step** **Step 4:** Chromosome Selection for the Next Iteration Create PPool by adding chromosomes from PCurr and PMod. Select 20 chromosomes from PPool using roulette wheel selection to form the new PCurr. **End Step** **Step 5:** Rectification of So Far Best Chromosome (SSO) Apply Sequential Search Operations (SSO) to fine-tune CrS FBest: -Change each bit from 1 to 0, check if it improves EA (ensemble accuracy). -Change each bit from 0 to 1, check if it improves EA. **End Step** **End for**

To increase ensemble accuracy, the suggested Opt-Forest Algorithm is an iterative optimization method for choosing and fine-tuning subforests inside a forest of trees. By blending good-quality tree prioritization with randomness, it carefully chooses chromosomes that represent subforests in each iteration. Crossover and mutation are two genetic processes that are used to create child subforests, and an elitist mechanism makes sure that the best-performing subforests are kept. In addition, the method uses a roulette wheel to choose the chromosomes for the subsequent iteration and sequential search techniques to improve the best subforest thus far. This iterative procedure continues for a predetermined number of iterations with the ultimate goal of identifying an ideal or nearly ideal subforest arrangement.

## 4 Research design

**Fig. 1:**
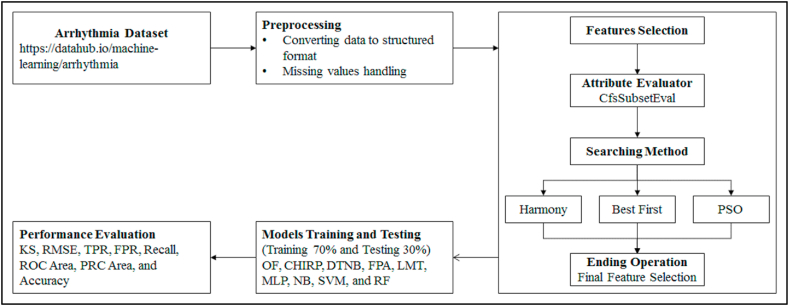
Overall Experimental Procedure of this Study.

This study focuses on enhancing CKD prediction with Opt-Forest using ECG signal data. The Opt-Forest models are compared with some standard ML models including CHIRP, DTNB, FPA, LMT, MLP, NB, SVM, and RF. These models are compared with Opt-Forest based on two different types of assessment measures which are KS and RMSE to measure the error rate of each model and TPR, FPR, accuracy, recall, ROC, and PRC areas for precision analysis. The overall procedure of this study is presented in Fig. 1, which is further discussed in the subsequent. The overall experiments are done using the system with Windows 10 operating system, 8 GB RAM with Core i7 processor. For experimentation of the models, Weka version 3.9.5 has been used.

### 4.1 Data acquisition and pre-processing

The dataset used in this study has been taken from Data Hub.^1^ The dataset includes features taken from the MIT-BIH Arrhythmia dataset (Physionet), which is a two-lead ECG signal (lead II, V). There are a total of 279 features in the dataset with sixteen classes. The classes are listed in Table 2.

**Table 2:** List of class attributes, descriptions, and number of entries.

Class	Description	Entries
Normal	Normal	245
IC-CAD	Ischemic changes (Coronary Artery Disease)	44
OAMI	Old Anterior Myocardial Infarction	15
OIMI	Old Inferior Myocardial Infarction	15
ST	Sinus tachycardy	13
SB	Sinus bradycardy	25
VPC	Ventricular Premature Contraction (PVC)	3
SPC	Supraventricular Premature Contraction	2
LBBB	Left bundle branch block	9
RBBB	Right bundle branch block	50
1-DAVB	1. degree Atrio-Ventricular block	0
2-DAVB	2. degree AV block	0
3-DAVB	3. degree AV block	0
LVH	Left ventricular hypertrophy	4
AF	Atrial Fibrillation or Flutter	5
Others	Others	22
**Total**		**452**

The preprocessing is divided into two steps. In the first step, the data was not in a structured format, we have converted all the data into a structured format. The second step involves missing the handling values. To do this, we have used replace missing values using the mean imputation method. It is a straightforward method for dealing with missing data is mean imputation, which involves substituting the missing values with the mean (average) of the observed values in the same feature (column). It frequently pertains to numerical data. The following equations can be used to mathematically describe mean imputation.

1. Calculate the Non-Missing Values' Mean (μ):

The mean of the observed (non-missing) values in the column must first be calculated. To do this, add up all the possible values, divide by the number of those values, and repeat:

(1) $$\mu =\frac{\sum_{i=1}^{n}x_{i}}{n}$$

μ is the mean, xi represents each observed value, and n is the count of observed values (excluding missing values).

2 Replacing the Missing Values with the Mean:

You may use this computed mean to fill in the missing numbers in the column after computing the mean:

(2) $$x_{\text{imputed}}=\mu$$

x_imputed_ are the imputed values for the missing data points.

### 4.2 Feature selection

The dataset initially had 279 features, making it exceedingly difficult to choose the most crucial ones. To do this, we used "CfsSubsetEval" as an attribute evaluator, which is frequently used when selecting features for ML or data mining activities. It assesses the importance of a set of traits (features) by taking into account both their predictive capability and the degree of overlap or redundancy among them. To prevent repetition, it is important to choose a subset of features that together offer valuable data for a predictive model. The process can be explained as.

• Individual Predictive Power (IP): Quantifies how well each feature predicts the target.
• Redundancy (R): Measures similarity or duplication between feature pairs, often using metrics like Pearson's correlation coefficient.

Given a feature subset S.

• Total Predictive Power (TP): The sum of individual predictive powers of features in S.

(3) TP(S)=∑IP(i)foralliinS

• Total Redundancy (TR): The sum of redundancies between pairs of features in S (excluding self-comparisons).

(4) TP(S)=∑∑R(i,j)foralli,jinS(i≠j)

A scoring function like the "Consistency Score" is used to balance TP and TR:

(5) $$\text{Consistency}\left(S\right)=\frac{\text{TP}\left(S\right)}{\sqrt{\left(\text{TR}\left(S\right)+\text{TP}\left(S\right)\right)}}$$

CfsSubsetEval uses search strategies like greedy forward selection or evolutionary algorithms to identify the subset S that maximizes the Consistency score. By using this technique, it is made sure that the chosen characteristics collectively provide the predictive model with useful information while reducing redundancy.

With the CfsSubsetEval, we use three different types of searching methods including Harmony Search (HS), Best First (BF), and PSO search. The three feature selection techniques were chosen to thoroughly examine and select the most useful ECG characteristics for CKD prediction. These techniques all offer different perspectives on feature selection. While iteratively enhancing feature subsets, HS introduces unpredictability; BF offers a methodical manner to add or delete features to optimize a scoring function; and PSO functions as a population-based optimization strategy, enabling the generation of dynamic feature subsets. By using these several techniques, the study hoped to guarantee a full examination of the feature space, select the most pertinent features for improved CKD prediction with ECG data while reducing duplication, and eventually improve the model's performance and accuracy.

a. **Harmony Search:** CfsSubsetEval and the heuristic optimization technique Harmony Search (HS) are used to choose the attributes. It iteratively improves random feature subsets at first. The Consistency Score (CS) (see Equation (5)), where TP(S) evaluates the ability to forecast, and TR(S) measures the amount of redundancy in subset S. New harmonies (feature subsets) are created by fusing previously existing ones in each cycle, with minor tweaks made to account for variation. If the best harmony raises the CS score, it is kept. This method continues until convergence requirements are satisfied, after a certain number of rounds. The algorithm ultimately chooses the feature subset with the greatest CS rating.
b. **Best First Search:** Using CfsSubsetEval and Best First (BF) Search, feature selection is improved by repeatedly adding or eliminating features to increase the CS:
  • Start with a feature subset S that is empty.
• Utilizing the goal function, determine CS(S).
• Examine the additions and deletions of features:
  o If a feature improves CS(S), add it.
  oIf a feature increases CS(S), remove it.
• Continue doing this until a halting requirement is satisfied.
  • The feature subset with the greatest CS score should be chosen.

TP(S) measures predictive power and TR(S) measures redundancy in the subset S as discussed in Equation (5).

**C. Particle Swarm Optimization Search:** By developing a population of feature subsets, Particle Swarm Optimization (PSO) for attribute selection in CfsSubsetEval operates. Particles (representing subsets) mathematically modify their locations (features) according to their velocity and the positions that are known to exist. TP and TR must be balanced to maximize the CS (see Equation (5)):

Particles update positions and velocities following:

(6) v_i(t+1)=w*v_i(t)+c1*rand1*(p_best_i−x_i(t))+c2*rand2*(g_best−x_i(t))

(7) x_i(t+1)=x_i(t)+v_i(t+1)

After iterations, select the feature subset with the highest CS score, optimizing attribute selection.

As indicated in Table 3, each of these search techniques identified distinct groups of features, and we then used the ending operation to choose the subset of features based on the majority vote approach. We only choose features that contain more information about the study's goal and have been chosen by two or all three searching techniques. This operation is mathematically represented as follows.

• Let F represent the collection of all possible features.
• F1, F2, and F3 are the feature subsets determined by the three distinct feature selection algorithms.
• F_selected_ is the final subset of features selected using the majority vote method.

**Table 3:** List of Selected Features using each Searching Method.

Search Method	Selected Features
Harmony Search	BI, BN, BY, DB, DK, EB, EF, EM, EN, FB, FC, FO, GR, HN, HR, IJ, IV, JB, JO, KO, KS, KU = **22**
Best First Search	qrs_duration, q-t_interval, t_interval, T, heart_rate, AU, CJ, DA, DD, DK, DN, DZ, EB, HJ, HR, IH, IN, IT, IV, JB, JV, JY, KS, LE, LG = **25**
PSO Search	qrs_duration, t_interval, r_wave, AH, BN, BO, BV, CJ, CZ, DK, DM, DO, DS, DZ, EB, EM, EY, FA, FT, GE, GO, HL, HR, HT, IN, JB, JD, JH, JJ, JO, JP, JV, JY, KH, KS, KY, LE = **37**
Ending Operation	qrs_duration, LE, JY, JV, JO, IN, EM, DZ, CJ, BN, IV, KS, JB, HR, EB, DK = **16**

The following is the mathematical equation for our ending operation:

(8) $$F_{\text{selected}}=\{f\in F||\{i\in \left\{1,2,3\right\}|f\in \text{Fi}\}|\geq 2\}$$

This equation chooses features from at least two of the three feature subsets indicated by your various feature selection methods. This is a majority vote method, in which characteristics chosen by a majority of the techniques are included in the final subset.

### 4.3 ML models, training, and performance evaluation

This study aims to use ECG signals to increase the precision of CKD prediction. The predicted models are contrasted with several standard ML models shown in Table 4. 70 % of the data are used to train each model, while the final 30 % are used for testing. Utilizing the standard evaluation metrics including TPR, FPR, recall, CA [5,6], ROC area, PRC area [7,8], KS [9], and RMSE [10,11], the performance evaluation is conducted. These metrics may be obtained using the confusion matrix generated by your ML model. The confusion matrix has four values: True Positives (TP), False Positives (FP), True Negatives (TN), and False Negatives (FN).

(9) $$\text{TPR}\;/\;\text{Recall}=\frac{\text{TP}}{\text{TP}+\text{FN}}$$

(10) $$\text{FPR}=\frac{\text{FP}}{\text{FP}+\text{FN}}$$

(11) $$\text{CA}=\frac{\text{TP}+\text{TN}}{\text{TP}+\text{FP}+\text{TN}+\text{FN}}$$

(12) $$\text{KS}=\frac{\text{Po}-\text{Pe}}{1-\text{Pe}}$$

In this equation, Po (Observed Agreement) is the proportion of situations in which the predicted and actual classifications match, and Pe (anticipated Agreement by Chance) is the proportion of cases in which agreement would be anticipated by chance.

(13) $$\text{RMSE}=\sqrt{\frac{1}{N}\;\sum_{i=1}^{N}{\left(y_{i}-\;{\hat{y}}_{i}\right)}^{2}}$$

In this case, N is the number of samples, y_i_ is the true (actual) label or value, and $\hat{y}$_I_ is the predicted label or value.

**Table 4:** List of the Models Employed in this Study Compared with the Projected Model.

ML Models	References
Hypercube on Iterated Random Projections	[1,12,13]
Decision Table-Naive Bayes	[[14], [15], [16]]
Forest by Penalizing Attributes	[10,12,17]
Logistic Model Tree	(M. Nikhil Kumar, K. V. S. Koushik, 2018; [10,18])
Multilayer Perceptron	[[19], [20], [21]]
Naïve Bayes	[21,22,22]
Support Vector Machine	[1,23,24]
Random Forest	[18,20,25]

**Receiver Operating Characteristic (ROC) Area:** The ROC area is computed by graphing TPR versus FPR at various decision thresholds using the ROC curve. The ROC area is often calculated using numerical integration or software library techniques.

**Precision-Recall Curve (PRC) Area:** The PRC area is derived by graphing Precision versus Recall at various decision thresholds using the PRC curve. The PRC area is commonly calculated using numerical integration or software library tools.

## 5 Results analysis and discussion

**Fig. 2:**
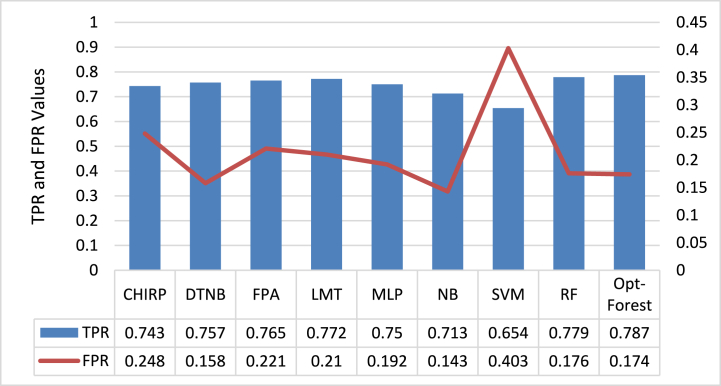
TPR and FPR Analysis of each Employed Compared with Opt-Forest Model.

Fig. 2 depicts a complete overview of the performance measures, including TPR and FPR, for a variety of ML models used in the context of CKD prediction utilizing ECG signal data. Opt-Forest is the most promising contender among the models studied. It has an astounding TPR of 0.787, indicating its capacity to successfully identify persons with CKD while displaying great sensitivity. Its low FPR of 0.174 also indicates its ability to minimize false alarms and not misclassify healthy persons as CKD-positive, resulting in good specificity. This sensitivity-specificity balance is especially important in medical diagnostics, where failure to detect true cases (false negatives) can have serious effects, while excessive false alarms can result in wasteful medical procedures. Opt-Forest's superior performance indicates that it excels at collecting significant patterns within ECG data suggestive of CKD, presumably due to its sophisticated ensemble methodologies and feature selection approaches. The most promising model for CKD prediction appears to be Opt-Forest, which demonstrates a compelling balance of accuracy and efficiency.

**Fig. 3:**
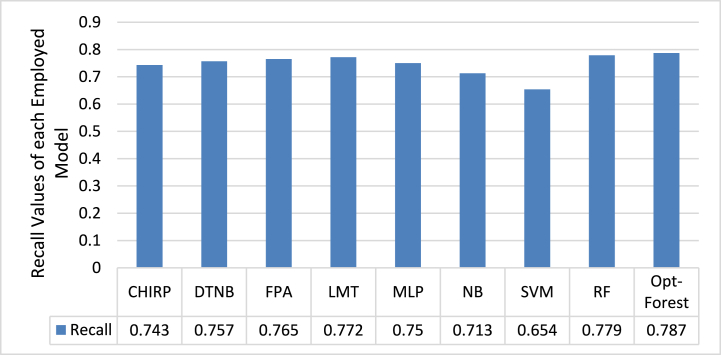
Analysis of each Employed Model through Recall Matric.

Fig. 3 shows the recall values for employed ML models, including Opt-Forest, that were used to predict CKD using ECG signal data. With a better recall score of 0.787, Opt-Forest significantly beats the other models. This demonstrates its better capacity to distinguish persons with CKD from those who do not have the illness. Opt-Forest's ensemble nature, as well as its feature selection procedures, most likely contribute to its good performance by successfully identifying key patterns within ECG data suggestive of CKD. SVM, on the other hand, looks to be the weakest model in this case, with a recall of 0.654. SVM's poor performance could be attributed to its decision boundary not adapting well to the complex and possibly non-linear relationships present in the ECG signal data, emphasizing the importance of selecting models that are well-suited to the specific characteristics of the dataset and the problem at hand.

**Fig. 4:**
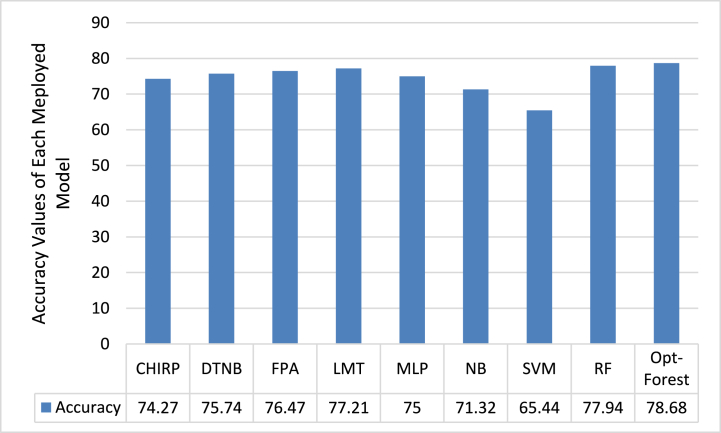
Accuracy Analysis of each Model used in this Study for CKD using ECG Signals.

Accordingly, the accuracy values are shown in Fig. 4. Opt-Forest performs significantly better than the other models, with an Accuracy score of 78.68 %. This shows that Opt-Forest is the best model for categorizing both CKD-positive and CKD-negative patients accurately. Its ensemble-based methodology, which combines the advantages of many decision trees and adds cutting-edge feature selection approaches, is probably responsible for its improved performance. Opt-Forest can capture the intricate correlations included in the ECG signal data in this way, increasing the classification accuracy on the whole. SVM, on the other hand, appears to be the least reliable model in this situation with an Accuracy of 65.44 %. The linear character of SVM, which might not be able to fully capture the complex patterns in the data, could be the cause of its worse performance. As the best performance overall in terms of accuracy, Opt-Forest demonstrates its applicability for CKD prediction with ECG signals.

**Fig. 5:**
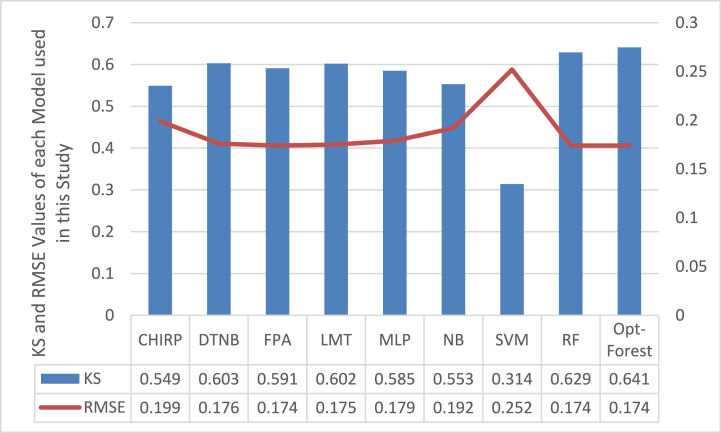
Error Rate Analysis of each Employed Model using KS and RMSE.

In the context of predicting CKD using ECG signal data, Fig. 5 shows the KS and RMSE values for employed ML models, including Opt-Forest. The best-performing model, Opt-Forest, stands out with the greatest KS (0.641) and lowest RMSE (0.174). The high KS suggests a significant amount of chance agreement correction between predicted and actual classifications, which is essential for precise CKD diagnosis. The model's accuracy in forecasting the probabilities linked to CKD is also shown by the model's low RMSE, which makes it a great choice for clinical decision support systems. Both KS and RMSE are crucial evaluation measures for classification jobs since they shed light on the model's functionality, agreement with the real world, and accuracy of its probability estimations. In this situation, Opt-Forest performs exceptionally well in both areas, making it a solid and trustworthy option for CKD prediction based on ECG data.

For several compelling reasons, Opt-Forest emerges as the best-performing model in this investigation for CKD prediction using ECG signal data. To efficiently collect and incorporate the complex patterns within ECG data suggestive of CKD, Opt-Forest first makes use of ensemble approaches and sophisticated feature selection algorithms. Its ensemble technique combines the advantages of many decision trees, improving its capacity to recognize important disease-related traits. Opt-Forest excels in terms of overall accuracy, sensitivity, and specificity. It has a high TPR, demonstrating its accuracy in detecting people with CKD, and a low FPR, reducing the possibility of false alarms. Diagnostics in medicine depend on this equilibrium. Additionally, Opt-Forest has remarkable KS and a low RMSE, demonstrating both good agreement with real classifications and accuracy in forecasting CKD probability. Its thorough methodology reveals its capacity to extract pertinent data from ECG signals for precise CKD prediction, including great sensitivity, specificity, and accuracy. Other models, however, can suffer because they can't handle intricate data linkages or because they use less sophisticated feature selection techniques. Overall, Opt-Forest's higher performance is due to its ensemble-based methodology, which successfully utilizes ECG data and leads to improved early CKD identification and management.

## 6 Conclusion

This study provides a thorough analysis of the Opt-Forest algorithm's utility in predicting CKD using ECG signal data. The study compared Opt-Forest with several well-known ML models frequently used in diagnoses of medical conditions. In terms of accuracy and error rate metrics, the findings show that Opt-Forest performs better than the other models, highlighting its superior capacity to extract useful data from ECG signals for accurate CKD prediction. Its impressive performance is a result of an ensemble-based method coupled with sophisticated feature selection algorithms, underscoring its potential as a useful tool for the early diagnosis of CKD and enhanced medical diagnostics. To improve patient outcomes through early treatment and diagnostic improvements, this research highlights the necessity of using ECG data to evaluate renal function and emphasizes the need for early CKD identification in clinical settings.

This research can be expanded in several ways in the future to improve the diagnosis and treatment of CKD. First off, adding more patient-specific data, such as demographics, medical history, and genetic markers, may increase the models' ability to predict outcomes accurately. Additionally, using neural networks and deep learning methods to fully harness the intricacy of ECG data may result in even more reliable CKD prediction models. Continuous CKD monitoring and early management might be facilitated by the combination of telemedicine platforms and real-time monitoring of ECG signals via wearable technology. Translating these findings into actual practice will need collaboration with healthcare organizations and doctors to verify the models on bigger and more varied patient groups. Last but not least, research into the possibility of individualized therapy suggestions based on CKD risk estimations might considerably enhance precision medicine in nephrology.

## CRediT authorship contribution statement

**Muhammad Binsawad:** Writing – original draft, Visualization, Validation, Methodology, Investigation, Formal analysis, Data curation, Conceptualization.

## Declaration of competing interest

The author declared no conflict of interest.

## Footnotes

1 https://datahub.io/machine-learning/arrhythmia.
